# Application of Reproductive Toxicity Caused by Endocrine Disruptors in Rotifers: A Review

**DOI:** 10.3390/biology15020128

**Published:** 2026-01-11

**Authors:** Guangyan Liang, Shenyu Liu, Shan Wang, Yuxue Qin

**Affiliations:** College of Marine Science and Technology and Environment, Dalian Ocean University, Dalian 116023, China; 13638146296@163.com (G.L.); 15711505696@163.com (S.L.)

**Keywords:** endocrine-disrupting chemicals, rotifers, reproductive toxicity, sensitivity

## Abstract

Endocrine-disrupting chemicals (EDCs), widespread in aquatic environments, interfere with endocrine function in organisms and threaten ecosystem stability. Rotifers, critical live feed for marine fish, shrimp, and crab larvae, link EDC-induced reproductive impairment to marine ecosystem stability and aquaculture sustainability. This PRISMA-compliant review synthesizes key findings, consequences, and gaps in EDC–rotifer reproductive toxicity research. Traditional EDCs (heavy metals, per- and polyfluoroalkyl substances (PFASs), phenols, phthalate esters, polybrominated diphenyl ethers (PBDEs), and steroid hormones) and emerging EDCs (disinfection byproducts, microplastics, pharmaceutical metabolites) induce distinct reproductive harm—e.g., Hg^2+^ shows extreme toxicity (24 h LC_50_ = 4.51 μg L^−1^ in *Brachionus plicatilis*), BDE-47 damages ovaries, and microplastics cause transgenerational delays. Rotifer species and exposure duration affect sensitivity (e.g., BDE-47: 96 h LC_50_ = 0.163 mg L^−1^ vs. 24 h LC_50_ > 22 mg L^−1^ in *B. plicatilis*). Oxidative stress is a universal mechanism, and combined EDC exposure produces context-dependent synergistic/antagonistic effects. EDC-induced impairment reduces rotifer population density, alters structure, and propagates through food webs, threatening aquaculture and biodiversity; transgenerational toxicity (e.g., 4-nonylphenol: F_1_ inhibition 28% vs. 12% in F_0_) weakens resilience. This review supports EDC risk assessment, with gaps including long-term low-concentration data, transgenerational mechanisms, EDC–microbiome interactions, and emerging PFAS toxicity—priorities for future research.

## Simple Summary

Endocrine-disrupting chemicals (substances that interfere with organisms’ hormone systems) are common in water environments and threaten ecosystems. Rotifers, key food for aquaculture larvae and important for aquatic balance, face reproductive harm from these chemicals. This study summarized how various chemicals (e.g., some plastics, heavy metals) affect rotifers’ reproduction, compared rotifer species’ sensitivity, and explored toxic mechanisms (e.g., oxidative stress, a process that causes cell damage). Results showed these chemicals reduce rotifer numbers and harm offspring. The study identifies research gaps and helps improve water quality standards, protecting aquaculture and ecosystems.

## 1. Introduction

Endocrine-disrupting chemicals (EDCs) are exogenous substances that can interfere with the synthesis, secretion, transport, or receptor binding of endogenous hormones. According to the TEDX database and the WHO/IPCS guidelines, over 1000 chemicals have been identified or proposed as potential EDCs [1]. These include persistent organic pollutants (e.g., polychlorinated biphenyls and dioxins), plastic additives (e.g., bisphenol A and phthalates), pesticide residues, and phenolic compounds found in personal care products [2,3]. By mimicking or antagonizing natural hormones, these substances disrupt endocrine homeostasis by interfering with hormone synthesis, metabolism, transport, and receptor-mediated signaling. This disruption ultimately leads to adverse effects on reproductive, skeletal, metabolic, and immune functions [4,5,6,7,8,9]. The primary sources of this issue include industrial pollution, municipal wastewater, domestic waste, agricultural runoff, food products, and personal care items [10,11,12]. They enter aquatic environments via wastewater discharge, soil leaching and other pathways during production, use and disposal. This infiltration leads to persistent environmental contamination [13,14]. Estrone, estradiol, bisphenol A (BPA), and nonylphenol are commonly detected in surface waters, albeit at trace levels. Their potential to disrupt endocrine functions presents significant risks to aquatic organisms [15]. Notably, many toxicological studies employ higher exposure concentrations than those typically found in the environment to rapidly observe distinct toxic effects. However, conducting investigations at environmentally relevant concentrations is crucial for accurately evaluating the risks associated with long-term low-dose exposures. Persistent organic pollutants (e.g., Polybrominated diphenyl ethers, PBDEs) and polyfluoroalkyl substances (PFASs) are persistent and liable to bioaccumulate. They may pose significant threats to aquatic organisms’ reproductive health via prolonged low-concentration exposure.

**Figure 1:**
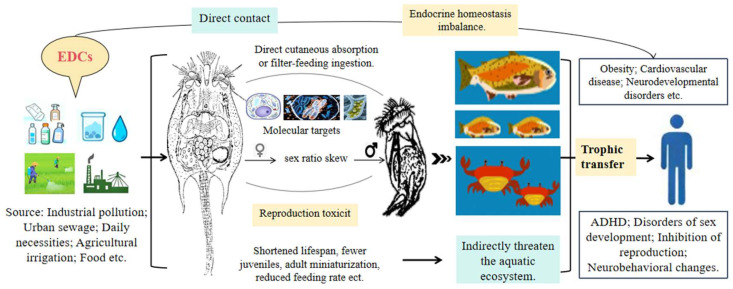
Environmental Fate and Human Health Impacts of EDCs.

Rotifers are widely used as live feed for fish, shrimp and crab larvae in aquaculture [16]. They serve as keystone species in aquatic ecosystems, occupying a fundamental position within the trophic hierarchy. The population dynamics of these organisms can be substantially influenced by energy transfer to higher trophic levels (Figure 1). Evidence indicates that exposure to EDCs induces reproductive toxicity in rotifers, which serve as a primary live feed for marine fish larvae. Impairment of rotifer reproduction may propagate through the food web, posing a significant threat to the sustainability of marine aquaculture and biodiversity [17,18]. Reduced rotifer populations or compromised reproductive capabilities can mitigate algal grazing pressure to some extent. Research indicates that rotifers play a vital role in regulating algal blooms, particularly those induced by cyanobacteria. A decline in their population due to pollutants (e.g., BPA) or environmental stressors may trigger a rapid resurgence of algae [19]. Notably, stress-induced overcompensation is not limited to EDCs but is prevalent across various stressors. For example, female rotifers under food limitation tend to overproduce offspring, leading to two associated fitness costs: reduced progeny size and decreased adult lifespan [20]. Rotifer populations play a crucial role in alleviating direct grazing pressure. Furthermore, fluctuations in these populations may have indirect effects on ecosystem stability. This influence is mediated through trophic cascades within the food web [21]. Studies have shown that certain EDCs (e.g., BPA) inhibit rotifer sexual reproduction by interfering with endocrine signaling pathways (e.g., estrogen receptor-mediated pathway). Steroid hormones (e.g., testosterone, estradiol) and their pollutant-derived derivatives interfere with rotifers’ endocrine systems. This disrupts the rotifers’ parthenogenetic cycle and triggers a shift to sexual reproduction (male/female production). Such reproductive mode changes typically reduce population density or alter population structure, posing potential risks to aquatic ecosystems [22,23,24,25]. Moreover, rotifers facilitate the trophic transfer of adsorbed microplastics and chemical contaminants. This transfer induces bioaccumulation in higher trophic organisms (e.g., fish, amphibians, mammals). These findings highlight that rotifer health is directly linked to the sustainability of marine aquaculture and the preservation of biodiversity. Furthermore, human EDC exposure via drinking water and personal care products may disrupt endocrine homeostasis. This disruption causes adverse health outcomes (e.g., obesity, cardiovascular diseases, attention deficit hyperactivity disorder (ADHD), sexual development abnormalities) [26,27].

Previous research has documented a range of adverse health effects in humans associated with EDCs, including metabolic disorders and neurodevelopmental impairments. Systematic assessments of reproductive toxicity across various classes of EDCs in aquatic ecosystems remain limited. This scarcity is particularly evident in basal trophic levels such as rotifers. Furthermore, the cascading ecological consequences of EDC-induced alterations in rotifer populations are understudied. Although numerous toxicity and mechanistic studies on EDCs (e.g., polybrominated diphenyl ethers, phthalates, heavy metals) have been conducted in rotifers [28,29,30], comprehensive systematic reviews of their reproductive toxicity remain lacking. Furthermore, most current toxicity assessments focus on responses in a single generation, thereby overlooking cumulative, adaptive, or transgenerational effects that may arise across multiple generations. The brief generation time of rotifers renders them an excellent model for exploring the multigenerational toxicity of EDCs. Clarifying the effects of parental generation exposure to EDCs on offspring fitness, reproductive strategies, and population resilience is essential for a thorough ecological risk assessment. To address this knowledge gap, the present study systematically reviews recent findings regarding EDC-induced reproductive toxicity in rotifers. It emphasizes interspecific variations in sensitivity, underlying toxicological mechanisms, and their implications for ecological risk management.

**Figure 2:**
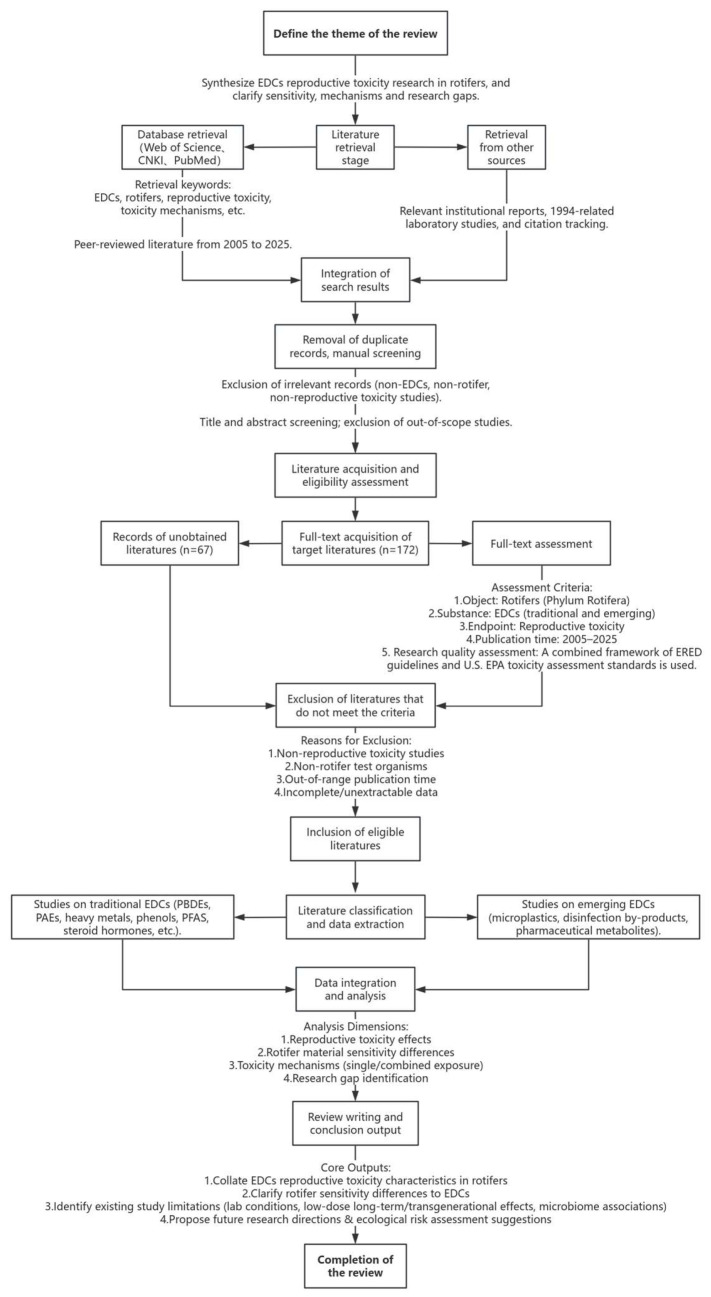
The PRISMA Flow Diagram.

This review adheres strictly to PRISMA guidelines, ensuring rigor, transparency, and critical evaluation. It employs a structured literature screening process, a multidimensional analytical framework, and transparent methodologies to identify research gaps and enhance the reliability of conclusions and the academic value of the findings [31]. This study systematically synthesizes existing research on the reproductive toxicity of EDCs in rotifers. It elucidates the toxic effects, highlights differences in sensitivity, explores mechanisms of action, and identifies gaps in current research. It specifies search databases (Web of Science, CNKI, PubMed, etc.), a time frame (2005–2025), and bilingual search term combinations. The sole exception is a seminal study conducted by Hagiwara Laboratory in 1994, which significantly contributes to the discourse on shifts in rotifer reproductive modes. This study delineates the inclusion and exclusion criteria for various rotifer species and the dimensions of reproductive toxicity. It employs a comprehensive four-step screening process that includes removing duplicates, automated preliminary screening, manual preliminary screening, and full-text assessment. This meticulous approach ensures the relevance and scientific rigor of the included literature and adheres to PRISMA transparency standards (Figure 2). We retrieved literature using the core keywords endocrine disruptors, EDCs, rotifers, reproductive toxicity, and toxicity mechanisms across both English and Chinese databases. Study quality was graded as High/Medium/Low using a combined framework of the CRED guidelines and EPA toxicity study assessment criteria. For reviewer conflicts, direct discussion was first attempted; if unresolved, a third independent reviewer with relevant expertise was consulted for arbitration. To further comply with the transparency requirements of the PRISMA guidelines, this section elaborates on the precise literature search strategy, the screening criteria for databases, and the decision criteria at each stage. The screening process is summarised in a table (Table 1) to enhance the rigour and reproducibility of the methodology.

## 2. Reproductive Toxicity of Different Endocrine-Disrupting Chemicals in Rotifers

### 2.1. Polybrominated Diphenyl Ethers

Polybrominated diphenyl ethers (PBDEs) are classified as persistent organic pollutants (POPs) [32,33]. These compounds disrupt endocrine pathways, including those involved in thyroid hormone, estrogen, and androgen signaling. Consequently, they can lead to thyroid dysfunction, impaired sexual development, and neurobehavioral alterations. As a result, PBDEs are categorized as EDCs [34,35,36]. Notable representative congeners include 2,2′,4,4′-tetrabromodiphenyl ether (BDE-47), 2,2′,3,4,4′,5′,6-heptabromodiphenyl ether (BDE-209), and decabromodiphenyl ethane (DBDPE). Marine rotifers have emerged as ideal model organisms for evaluating chronic reproductive toxicity and elucidating the underlying mechanisms associated with PBDE exposure. Ultrastructural analyses demonstrate that BDE-47 induces severe ovarian damage. The 48 h, 72 h, and 96 h median lethal concentrations (LC_50_) of BDE-47 are 2.113 mg L^−1^, 0.376 mg L^−1^, and 0.163 mg L^−1^, respectively, versus 11.162 mg L^−1^, 1.237 mg L^−1^, and 0.295 mg L^−1^ for BDE-209 [37]. Both BDE-47 and BDE-209 exhibit a notable time-dependent decrease in LC_50_ with extended exposure, suggesting that toxicity increases over time. This observation underscores the limitations of risk assessments that rely solely on acute toxicity data. In natural environments, factors such as temperature, pH, and other parameters can further enhance PBDE bioaccumulation and toxicity. Consequently, it is essential to incorporate characteristics of chronic exposure into the development of more stringent risk management standards. In the 24-h acute toxicity assay, BDE-209 exhibited no acute lethality, even at the maximum tested concentration of 120 mg L^−1^. In contrast, BDE-47 at a concentration of 22 mg L^−1^ resulted in less than 50% mortality in *Brachionus plicatilis* [38]. For *Brachionus koreanus*, exposure to BDE-47 significantly reduces lifespan and inhibits reproductive capabilities [39]. PBDEs significantly inhibit reproductive rates and egg production in rotifers. Liu et al. reported that concentrations of 0.5–1 µg L^−1^ of BDE-47 reduce the daily egg output of female rotifers, while BDE-209 exhibits inhibitory effects at slightly higher concentrations (2–5 µg L^−1^) [38]. Co-exposure to BDE-47 and DBDPE induces either antagonistic or additive effects on reproductive duration, offspring production, and survival in well-nourished *B. plicatilis* [40]. Long-term exposure (21 d) to 0.1 μg L^−1^ of BDE-47 significantly diminished the growth rate of rotifer populations. This concentration aligns with reported levels found in aquatic environments impacted by industrial wastewater, suggesting a non-negligible ecological risk. In contrast, the toxic effects of BDE-209 become evident only at higher concentrations. This discrepancy may be attributed to variations in environmental migration, transformation, and bioavailability among different PBDEs [41]. Zhang et al. conducted a further investigation into the effects of PBDEs on the hatching success and embryonic development of *B. plicatilis*. Exposure to BDE-47 resulted in a reduction in hatching rates by 15–25% within 48 h, alongside an increase in the rates of embryonic malformations. In contrast, BDE-209 primarily caused delayed hatching, extending the duration by 12–18 h. Gene expression analysis revealed that BDE-47 significantly downregulates mRNA levels of key reproductive genes (e.g., *vasa* and *nanos*), whereas BDE-209 exhibits only mild inhibition, detectable only at elevated concentrations [41].

Compared to BDE-209, BDE-47 exhibits greater lipophilicity and demonstrates a higher propensity for accumulation in lipid-rich tissues, such as rotifer ovaries. It significantly elevates reactive oxygen species (ROS) levels, induces lipid peroxidation and DNA damage, and activates the lysosomal apoptosis pathway by reducing mitochondrial membrane potential. Furthermore, it inhibits key reproductive genes while robustly stimulating the mitogen-activated protein kinase (MAPK) stress pathway. This multi-pathway interaction underlies the more pronounced reproductive toxicity of BDE-47 at equivalent concentrations. In contrast, BDE-209 is characterized by low aqueous solubility and bioaccumulation efficiency, exhibiting minimal ROS-inducing and MAPK-activating effects [39,42,43]. Consequently, it necessitates substantially higher doses to initiate toxic pathways, resulting in limited interference with reproductive functions.

### 2.2. Phthalate Esters

Phthalates (PAEs), a typical class of endocrine disruptors, can disrupt the stability of sex hormones by mimicking or antagonizing endogenous hormones. Their endocrine-disrupting effects have been well-verified in mammalian models. For instance, they can lead to abnormal gonadal function, decreased reproductive performance, and transgenerational reproductive damage through mechanisms such as inhibition of fetal testosterone synthesis, induction of oxidative stress, and disruption of estrogen and androgen receptor signaling pathways [44,45,46]. However, direct research on the reproductive toxicity of PAEs on rotifers is still minimal. The current understanding mainly relies on indirect inferences from mammalian studies, lacking targeted experimental data based on the unique physiological characteristics of rotifers, such as their alternating asexual and sexual reproductive patterns and the endocrine regulation mechanisms specific to invertebrates. The few existing studies on rotifers mainly focus on di(2-ethylhexyl) phthalate (DEHP), dibutyl phthalate (DBP), and butyl benzyl phthalate (BBP), and are mostly acute toxicity or single-effect observations.

Systematic reviews have demonstrated that PAEs exert reproductive toxicity in mammals. This occurs mainly by inhibiting fetal testosterone synthesis, inducing oxidative stress, and disrupting estrogen and androgen receptor signaling pathways [47]. The 24-h LC_50_ for DBP in *B. plicatilis* is 8.02 mg L^−1^, compared to 12 mg L^−1^ for BBP. Both compounds interfere with lipid metabolism, damage ovarian structure, induce apoptosis, generate oxidative stress, and cause DNA damage in *B. plicatilis*, ultimately diminishing its reproductive capacity [48,49]. Like *B. plicatilis*, *B. koreanus* exhibits significant reproductive inhibition in response to PAEs exposure, a phenomenon often linked to dysregulation of the antioxidant system [50]. Cruciani et al. discovered that three PAEs—DBP, BBP, and DEHP—exert dose-dependent effects on the sexual reproduction of *Brachionus calyciflorus*. Specifically, DBP at concentrations ≥0.5 mg L^−1^ reduces both population and intrinsic growth rates. In contrast, low concentrations of BBP (ranging from 0.05 to 0.5 µg L^−1^) stimulate population growth; however, higher concentrations of BBP (from 0.5 to 500 µg L^−1^) inhibit growth and resting egg production. Furthermore, moderate concentrations of DEHP (between 0.005 and 500 µg L^−1^) promote the production of resting eggs. In contrast, high concentrations of DEHP (at 5000 µg L^−1^) induce reproductive toxicity by impairing critical reproductive parameters such as fertilization rate [51].

Although the above-mentioned studies have initially revealed the potential reproductive hazards of PAEs to rotifers, they have obvious limitations: first, the research species are limited to the genus *Brachionus*, lacking toxicity data for other common rotifers (e.g., the genera *Asplanchna* and *Synura*), which cannot reflect the sensitivity differences in different rotifer groups to PAEs; second, the effect indicators are fragmented, with a focus on macroscopic indicators such as population growth rate and egg production, while there is a lack of research on microscopic mechanisms such as the development of reproductive cells (e.g., oocyte maturation and sperm motility) and the expression regulation of reproductive-related genes (e.g., *vasa* and *nanos*); third, the exposure conditions are disconnected from the actual environment, as most existing experiments use high concentrations (mg L^−1^ level) exposure, while the PAE concentrations in natural water bodies are primarily at the ng L^−1^ to μg L^−1^ level, and the chronic effects of long-term low-concentration exposure on rotifer reproduction and the transgenerational transmission effects remain unclear; fourth, the mechanism of action is not precise, although it is speculated that oxidative stress and endoplasmic reticulum stress are the key pathways by which PAEs inhibit rotifer reproduction, the specific molecular regulatory network (e.g., which signal molecules mediate the stress response and how they interact with endocrine signals) remains blank.

Given the widespread presence of PAEs in water environments and their potential threat to the basic trophic levels of ecosystems, it is urgent to carry out more specialized research on rotifers: It is suggested that the scope of research species be expanded as a priority, covering different trophic-level-dependent rotifer groups in freshwater and marine ecosystems; establish a research framework of “environmental concentration-multi-generation exposure-multi-level effects at molecular/cellular/population”, focusing on analyzing the long-term impact of low-concentration PAEs on the reproductive mode transformation, diapause egg formation and hatching of rotifers; combine transcriptomics, metabolomics and other omics technologies to clarify the specific molecular pathways by which PAEs interfere with rotifer reproduction, filling the gap in toxicity mechanisms between invertebrates and mammals; at the same time, carry out joint toxicity studies of PAEs with other pollutants (e.g., microplastics, heavy metals) to more realistically reflect the ecological risks of PAEs to rotifers in complex environments.

### 2.3. Heavy Metals

Recent research on the reproductive toxicity of heavy metals in rotifers has predominantly concentrated on copper (Cu), cadmium (Cd), zinc (Zn), chromium (Cr), manganese (Mn), lead (Pb), and mercury (Hg). For silver nanoparticles (Ag NPs), the 24-h and 48-h LC_50_ values in *B. plicatilis* are reported as 18.7 mg L^−1^ and 3.4 mg L^−1^, respectively. Ag NPs have been shown to reduce neonatal body length in both F_0_ and F_1_ generations, indicating adverse developmental effects during the non-fertilized egg stage in subsequent generations [52]. Acute toxicity assessments of Pb^2+^, Cd^2+^ and Hg^2+^ on *B. plicatilis* revealed 24 h LC_50_ values of 2.83 mg L^−1^, 2.07 mg L^−1^ and 4.51 μg L^−1^, respectively, indicating the species’ highest tolerance to Pb^2+^ and highest sensitivity to Hg^2+^ [53]. *B. calyciflorus* is widely used to evaluate the combined toxicity of mixed pollutants (e.g., copper + microcystins). Xu et al. determined the 24 h LC_50_ values of Cu^2+^, Zn^2+^, Cd^2+^, Cr^6+^ and Mn^2+^ for *B. calyciflorus* as 6.2 μg L^−1^, 12.62 mg L^−1^, 2.89 mg L^−1^, 17.29 mg L^−1^ and 67.32 mg L^−1^, respectively. Mechanistic analyses showed Cu^2+^, Cd^2+^ and Cr^6+^ induce cell death via severe oxidative stress and metal-sulfur complex formation, while Zn^2+^ and Mn^2+^ exert toxicity mainly through enzyme inhibition and mild oxidative stress. Multi-metal co-exposure aggravates oxidative stress and causes simultaneous damage to cell membranes, DNA, and mitochondria. This finding provides a theoretical basis for environmental risk assessment and management of mixed-metal pollution. It underscores the need to consider metal ratios rather than individual concentrations in water quality monitoring [54]. Additionally, research has shown that elevated temperatures and methylmercury exposure adversely affect rotifer survival, lifespan, and population dynamics. The processes of bioaccumulation, oxidative stress responses, and biochemical alterations are developmentally dependent, with neonates exhibiting greater susceptibility than adults [55]. This stage-specific vulnerability suggests that toxic effects may be transmitted maternally to offspring, thereby posing transgenerational risks.

Existing studies indicate that mercury and copper are the most toxic metals to rotifers, causing significant impairments in population survival and reproduction under elevated temperatures or chronic low-concentration exposure. Furthermore, heavy metals (e.g., copper, silver, and zinc) exhibit pronounced transgenerational toxicity, reproductive inhibition, and behavioral alterations in rotifers.

### 2.4. Phenols

Phenols are endocrine-disrupting chemicals characterized by the presence of phenolic hydroxyl groups, which interfere with the normal functioning of organisms’ endocrine systems. They are primarily classified into three categories: chlorophenols, alkylphenols, and bisphenols. Ubiquitous in various environmental contexts, phenols present potential hazards to animals, plants, and humans alike [56]. Phenolic EDCs—particularly nonylphenol (NP) and its derivatives—pose significant ecological risks due to their pervasive presence in aquatic ecosystems [57]. *Brachionus havanaensis* and *Plationus patulus* were selected as indicator organisms for emerging contaminants in a comparative study. Gómez et al. performed a multi-generational experiment to determine the 4-nonylphenol (4-NP) LC_50_ for two species. Results showed 4-NP significantly threatened their population demography, and while *B. havanaensis* had marginally lower acute toxicity than *P. patulus*. The F_1_ generation experienced a more severe population decline than the F_0_ in chronic toxicity tests [58]. This study confirms that the effects of long-term pollutant exposure are magnified in offspring, with multi-generational impacts proving to be more severe than those observed from single-generation exposure. Furthermore, marine rotifers exhibit a unique vulnerability to phenolic derivatives. Specifically, *B. koreanus* demonstrates pronounced sensitivity to these compounds, as evidenced by triclosan (TCS) and triclocarban (TCC) elevating reactive ROS levels and impairing cell membrane integrity in this species [59]. Recent studies show that BPA disrupts rotifer swimming behavior and nerve conduction, with *B. plicatilis* exposed to BPA exhibiting marked motor impairment, loss of directional control, and neuromuscular dysfunction [60]. Conversely, BPA induces reproductive and developmental toxicity in *B. calyciflorus* [61]. These findings confirm that phenolic compounds pose insidious, cumulative hazards to rotifers. They exert reproductive and neurotoxicity by interfering with the organisms’ endocrine signaling pathways.

### 2.5. Perfluorinated and Polyfluoroalkyl Substances

Recent research on the reproductive toxicity of per- and polyfluoroalkyl substances (PFASs) in freshwater rotifers has steadily increased. These studies specifically concentrate on perfluorooctanesulfonic acid (PFOS) and perfluorooctanoic acid (PFOA), utilizing *B. calyciflorus* as a representative species. Zhang et al. determined the 24 h LC_50_ values of PFOS and PFOA using *B. calyciflorus*, reporting 61.8 mg L^−1^ and 150.0 mg L^−1^, respectively. Long-term exposure reduces the species’ population growth rate (*r*) and resting egg production [62]. PFOS exposure alone significantly reduces population growth within two days, indicating that sublethal concentrations impair overall reproductive fitness [62]. Moreover, 96 h exposure to both PFOS and PFOA decreases the induction of mictic females and resting egg formation. These effects imply that PFASs exert reproductive toxicity via multiple mechanisms, including interference with sex hormone synthesis, induction of oxidative stress in germ cells, and disruption of reproductive-related local signaling pathways in rotifers [63,64]. Notably, while environmental concentrations of PFOS and PFOA typically remain below the mg L^−1^ threshold, research indicates that levels as low as 80 μg L^−1^ can significantly inhibit rotifer growth. Given the extreme environmental persistence and bioaccumulation potential of PFAS compounds, prolonged exposure to low doses exerts a sustained pressure on the population sustainability of essential forage organisms, such as rotifers [65]. The draft of the 2024 US EPA water quality criteria reaffirms the acute and chronic toxicity of PFOS to rotifers, identifying them as highly sensitive freshwater taxa. It recommends a 24-h LC_50_ value of approximately 62 mg L^−1^ as the scientific basis for establishing environmental thresholds [66]. The report underscores that PFAS concentrations in water are generally below the milligram per liter (mg L^−1^) level. However, their persistence and potential for bioaccumulation may result in cumulative adverse effects, thereby posing a significant risk to rotifer populations under prolonged exposure to low concentrations.

This study elucidates the acute and chronic reproductive toxicity of traditional PFASs, specifically PFOA and PFOS, on rotifers. It is essential to acknowledge the increasing prevalence of emerging PFAS alternatives, such as GenX, Potassium 9-chlorohexadecafluoro-3-oxanonane-1-sulfonate (F-53B), and 6:2 fluorotelomer alkyltrimethylammonium salt [67]. Although designed to have lower environmental persistence than PFOA, these alternatives may still produce significant biological effects. For example, exposure to GenX has been shown to induce neurotoxicity and oxidative stress in zebrafish embryos [68]. The transcriptomic analyses indicate that it regulates mitochondrial function and metabolic activity across various species [69]. Similarly, both 4,8-Dioxa-3H-perfluorononanoic acid (ADONA) and F-53B have been detected in various environmental media and exhibit comparable toxicity profiles in aquatic organisms [70,71]. However, data concerning the specific effects of these two substances (ADONA and F-53B) on rotifers remains exceedingly limited. These molecular-level interactions imply a mechanism of action that is analogous to that of PFOA [72]. These novel PFAS compounds may interfere with the energy allocation processes essential for rotifer reproduction. Although their solubility differences result in lower direct acute risks, these emerging endocrine disruptors currently lack species-specific impact data concerning rotifers. Therefore, urgent ecotoxicological screening is imperative to mitigate their potential adverse effects on aquatic ecosystems.

GenX and F-53B are low-persistence alternatives to traditional perfluorinated compounds such as PFOA and PFOS. Although they have shown specific toxic effects in model organisms such as zebrafish, their toxicological effects on rotifers remain largely unexplored. The current knowledge gap mainly lies in four aspects: Firstly, there is a lack of basic toxicity thresholds. The 24 h and 96 h LC_50_ values of GenX and F-53B for common rotifer species such as *B. plicatilis* and *B. calyciflorus*, as well as key ecotoxicological indicators like population growth rate and egg production, have not been obtained, making it impossible to effectively compare them with traditional PFASs (e.g., the 24 h LC_50_ of PFOS for *B. calyciflorus* is 61.8 mg L^−1^) (Table 2). Secondly, the reproductive toxicity and its mechanism remain unclear. There is a lack of data on their effects on reproductive mode transitions, diapause egg formation, and the hatching process of rotifers, and no research on the regulation of expression of key reproductive-related genes such as *vasa* and *nanos*. Thirdly, the combined effects of exposure and environmental relevance are insufficient. There have been no studies on the combined toxicity of GenX and F-53B in the presence of coexisting pollutants such as microplastics and heavy metals, and most existing studies ignore long-term low-dose exposure assessment within the actual environmental concentration range (ng L^−1^ to μg L^−1^). Fourthly, there is a lack of research on transgenerational toxicity. The cumulative effects on multiple generations of rotifers, the decline in reproductive capacity, and the potential transgenerational transmission mechanism of GenX and F-53B remain unknown.

### 2.6. Steroid Hormones

Acute toxicity data regarding the effects of steroid hormones on rotifers remain limited. Recent ecotoxicological studies indicate that the core toxicity data for these compounds primarily focus on 17β-estradiol (E2) and testosterone. Notably, E2 significantly inhibits both the population growth rate (r) and the mitotic rate of *B. calyciflorus* [83]. Testosterone exhibits significant reproductive toxicity in the freshwater rotifer *B. calyciflorus*. Tian et al. (2017) reported a 24-h LC_50_ of 10 µg L^−1^ for testosterone in this species; significantly, while testosterone does not induce immediate mortality in rotifers, it disrupts population dynamics by interfering with their sexual reproduction cycle [84]. Studies indicate that exogenous E2 significantly enhances the proportion of mictic females within *B. plicatilis* populations. Specifically, it mimics endogenous hormone signaling, activates edh gene expression, promotes estradiol synthesis, and induces the production of resting eggs [85]. Current research on combined exposure to steroid hormones primarily focuses on the mixture of ethinylestradiol (ETE) and levonorgestrel (LEV), specifically in relation to *Anuraeopsis fissa* and *B. calyciflorus*. Studies indicate that even at the lowest concentration of this hormone mixture, there is a significant inhibition of *A. fissa* population growth, highlighting its extreme sensitivity and vulnerability to low-dose physiological disturbances. In contrast, a relatively higher concentration of the hormone mixture is required to affect the fecundity and population growth rate of *B. calyciflorus*, as this species exhibits a more subdued acute toxic response than *A. fissa* [86]. These findings confirm that steroid hormone mixtures can disrupt the normal reproductive and developmental processes of rotifers.

### 2.7. Other Endocrine-Disrupting Chemicals

Beyond the aforementioned EDCs, physical radiation, emerging contaminants, and pharmaceutical metabolites also have the potential to disrupt endocrine function. In recent years, emerging contaminants have garnered significant attention within ecotoxicology research. These contaminants encompass nanomaterials, microplastics and their derivatives, disinfection byproducts, and pharmaceutical metabolites. However, relevant toxicity studies concerning rotifers remain relatively limited.

#### 2.7.1. Physical Radiation (Taking Ultraviolet B as an Example)

Investigations into the sensitivity of rotifers to physical environmental factors have predominantly concentrated on ultraviolet (UV) radiation; existing studies indicate that Ultraviolet B (UV-B) exposure induces acute lethality and compromises the antioxidant defense systems of rotifers when exceeding a specific intensity threshold [78,87]. Early studies demonstrated that the 24 h, 48 h and 96 h median lethal doses (LD_50_) of UV-B radiation for *B. plicatilis* are 4.393, 2.694 and 1.720 kJ/m^2^, respectively, compared with 5.856, 4.516 and 1.730 kJ/m^2^ for *Brachionus urceus* [79]. UV-B radiation significantly inhibits the population growth of *B. asplanchnoidis* and *B. urceolaris*, induces the expression of antioxidant enzymes (e.g., SOD and CAT) to alleviate oxidative stress, and leads to DNA damage in these rotifers [88].

#### 2.7.2. Toxicity and Reproductive Effects of Typical Emerging Pollutants (Microplastics, Nanomaterials, Disinfection By-Products) on Rotifers

Bromate, a prevalent disinfection byproduct found in drinking water, exhibits complex synergistic toxicity when interacting with polystyrene nanoplastics (PSNPs). These nanoplastics serve as carriers that enhance the bioaccumulation and toxicity of bromate in *B. calyciflorus* [89]. Yeo et al. demonstrated that maternal exposure to *B. koreanus* nanoplastics facilitates transfer of these nanoplastics to offspring via eggs, leading to developmental delays and reduced reproductive rates [90]. This provides direct confirmation that nanoplastics induce transgenerational toxicity through reproductive mediation, thereby impairing the developmental and reproductive performance of offspring. Fragmented polyethylene terephthalate (PET) particles exhibit no significant acute lethal toxicity to *B. koreanus*. However, prolonged exposure adversely affects its energy metabolism and disrupts the antioxidant system [50]. ZnO/TiO_2_ nanoparticles, utilized as photocatalysts or industrial additives, primarily demonstrate synergistic effects on *Proales similis*, with only a limited number of instances exhibiting antagonism or additivity [91]. Haloacetamides are recognized as endocrine-disrupting chemicals. Typical examples, such as bromoacetamide, chloroacetamide, and dichloroacetamide, have been shown to induce cellular structural damage and physiological dysfunction in *B. calyciflorus*. In response to this environmental stress, rotifers transition from amictic parthenogenesis to gonochorism in order to produce resting eggs that exhibit enhanced tolerance [80]. Snell et al. identified that a protein signal, Micticin, triggers the switch from asexual to sexual reproduction in rotifers. The 17-amino-acid N-terminal sequence of this protein exhibits high homology with a steroidogenic-inducing protein isolated from human ovarian follicular fluid. Upon reaching a specific concentration threshold, this protein prompts female rotifers to produce males or resting eggs, thereby elucidating the abrupt reproductive shift observed in rotifer populations under conditions of high density or resource limitation [92]. Conversely, Hagiwara et al. demonstrated that the external microbial environment influences rotifer reproduction through chemical signaling. The supplementation of rotifer cultures with specific bacteria (e.g., Pseudomonas) or rotifer extracts significantly disrupts asexual reproduction, thereby triggering sexual reproduction and the production of resting eggs [93]. This discovery highlights the extraordinary adaptability of rotifers in their response to environmental stressors.

#### 2.7.3. Drugs and Their Metabolites

For most organisms, the drugs themselves are seldom the primary source of risk. Instead, their metabolic transformation products play a crucial role, as these compounds can disrupt endocrine systems and induce various pathologies. Current toxicity studies on rotifers have primarily concentrated on the metabolites of carbamazepine, paracetamol, antibiotics, and antifungal agents. Notably, 10,11-epoxide—the principal active metabolite of carbamazepine—exhibits biological activity and possesses greater neurotoxic potential than its parent compound [94]. Early research has shown that the antiepileptic medication carbamazepine, along with its metabolites, inhibits acetylcholinesterase (AChE) activity in *B. koreanus*. This inhibition disrupts the normal functioning of its nervous system [95]. At elevated concentrations, paracetamol (APAP) and oxytetracycline (OTC) have the potential to inhibit the reproduction of *Brachionus rotundiformis* [81]. Antibiotics and antifungal agents, such as triphenyltin chloride (TPTCl), typically persist in the environment as drug residues and exhibit specific transmembrane toxicity to *B. koreanus* [82].

Current research on the reproductive toxicity of EDCs degradation products in rotifers remains inadequate. Given their observed endocrine-disrupting activity in mammalian models, it is reasonable to hypothesize that these degradation products may have detrimental effects on rotifers. Such effects could potentially target male reproductive systems and resting eggs through similar toxic mechanisms, including oxidative stress. Future studies should prioritize the identification and quantification of these degradation products, as well as the assessment of their combined toxic effects with the parent compounds.

## 3. The Sensitivity of Rotifers to Different Endocrine Disruptors

Rotifers are distinguished by their brief life cycles and heightened sensitivity to environmental pollutants. They are widely acknowledged as crucial indicator species for evaluating the ecotoxicity of endocrine-disrupting substances [96]. Their toxic effects can be quantified using LC_50_ and LD_50_ values. LC_50_ is primarily used in studies involving aquatic organisms, whereas LD_50_ serves as a fundamental benchmark for assessing chemical risks to mammalian health, including human health [97]. Generally, lower LC_50_ and LD_50_ values indicate heightened sensitivity of rotifers to target chemicals and increased corresponding ecotoxicity [98,99]. Based on these two toxicity metrics, we elucidated interspecific variation in rotifer sensitivity to EDCs.

Based on the 24-h LC_50_ data for various endocrine-disrupting chemicals (Table 2), the order of toxic sensitivity of *B. plicatilis* to these substances, from highest to lowest, was as follows: Hg^2+^ > Cd^2+^ > Pb^2+^ > BBP > Ag^+^ > BDE-47 (or BPA) > BDE-209. Notably, its sensitivity to most of these compounds increased significantly with prolonged exposure time. For instance, the 96-h LC_50_ values of BDE-47 and BDE-209 decreased by two orders of magnitude relative to the 24-h values (Table 2). However, after 96 h of exposure, the corresponding LC_50_ values decreased dramatically to 0.163 mg L^−1^ and 0.295 mg L^−1^. This indicates that sensitivity orders derived from short-term exposure do not accurately reflect those under long-term exposure conditions. The toxic sensitivity sequence of *B. calyciflorus* in response to these endocrine-disrupting chemicals (from high to low) was as follows: Cu^2+^ > C_2_H_3_BrNO > DBP > C_2_H_4_ClNO > Zn^2+^ > BPA > Cr^6+^ > PFOS > Mn^2+^ > PFOA > C_2_H_3_Cl_2_NO. For *B. koreanus*, the order of sensitivity was observed as follows: TPTCL > TCC > TCS > BDE-47. In contrast, *B. rotundiformis* exhibited notable differences in sensitivity to various EDCs. The 96-h LC_50_ values for APAP and OTC remained undetermined, exceeding 5000 mg L^−1^ and 200 mg L^−1^, respectively. Regarding 4-NP, the present study focused exclusively on two rotifer species: *B. havanaensis* and *P. patulus*. The sensitivity order observed over 24 h was as follows: *B. havanaensis* exhibited greater sensitivity at 250 µg L^−1^ than *P. patulus* at 500 µg L^−1^.

The LD_50_ applies to water-insoluble physical stressors. In Table 2, it was exclusively utilized for UV-B radiation (unit: kJ/m^2^). Lower LD_50_ values indicate greater sensitivity of rotifers to UV-B radiation. At 24 h of exposure, the order of UV-B sensitivity among rotifer species (from high to low) was as follows: *B. urceolaris* (5.856 kJ/m^2^) > *B. plicatilis* (4.393 kJ/m^2^) > *B. asplanchnoidis* (28.53 kJ/m^2^). The most sensitive species exhibited an LD_50_ at 24 h that was more than four times lower than that of the least sensitive species. Furthermore, rotifer sensitivity to UV-B radiation increased significantly with prolonged exposure. The 96-h LD_50_ values for *B. urceolaris* and *B. plicatilis* decreased to 1.730 kJ/m^2^ and 1.720 kJ/m^2^, respectively, indicating a convergence in sensitivity between these two species. The sensitivity rankings presented herein illustrate the varying risks posed by the assessed stressors to rotifer populations. This information is essential for conducting aquatic ecological risk assessments and formulating effective pollution control strategies.

We further compared the LC_50_ values of rotifers with environmental monitoring concentrations to calculate the hazard quotient (HQ = C_env_/LC_50_). Here, C_env_ denotes the environmental exposure concentration, and LC_50_ denotes the median lethal concentration. An HQ greater than 1 indicates an unacceptable level of risk and facilitates a quantitative risk assessment for small aquatic invertebrates [100]. This analysis has revealed distinct risk profiles across various pollutant categories. Heavy metals, such as Hg^2+^, exhibit extreme acute toxicity, with a 24-h LC_50_ for *B. plicatilis* measured at 4.51 μg L^−1^. Environmental concentrations at the μg L^−1^ level yield hazard quotient (HQ) values that approach critical thresholds. In contrast, organic EDCs, including BDE-47, BPA, and PFOS, demonstrate a characteristic of “low HQ but high chronic risk.” For instance, BDE-47 has a 96-h LC_50_ of 0.163 mg L^−1^; however, prolonged exposure to its environmentally relevant concentration of 0.1 μg L^−1^ (with an HQ of approximately 0.0006) significantly inhibits rotifer population growth through endocrine disruption—a detrimental effect obscured by the chemical’s low acute HQ value. PFOS presents a similar scenario, with a 24-h LC_50_ of approximately 62 mg L^−1^ and suppression of rotifer populations at concentrations ≤80 μg L^−1^ (HQ ≈ 0.0013). Its notable persistence and potential for bioaccumulation create sustained pressure from low-dose exposures over time. Consequently, relying solely on the acute LC_50_-based HQ framework substantially underestimates the ecological risks associated with these compounds. In summary, effective priority pollutant screening necessitates a dual-track strategy: one that regulates acutely toxic substances like mercury while also prioritizing organic compounds such as BDE-47, BPA, and PFOS—substances known to induce chronic reproductive disruptions in rotifers even at environmentally relevant concentrations despite their low acute HQ values. These compounds pose significant threats to both population sustainability and food web stability; thus, highlighting the imperative need to incorporate chronic effects alongside multigenerational and mixture-toxicity assessments into future risk evaluations.

## 4. Comparative Analysis of Acute Toxicity Criteria and Chronic Low-Dose Endocrine Disrupting Effects

The mg L^−1^-level concentrations used in acute toxicity tests (e.g., the 24 h LC_50_ of BDE-47 on *B. plicatilis* is 22 mg L^−1^, and that of BBP on the same species is 12 mg L^−1^) can quickly define the lethal thresholds of pollutants and clarify toxic mechanisms (e.g., inducing lysosomal rupture and activating the MAPK pathway) [37,39]. However, they are significantly disconnected from the ng L^−1^-μg L^−1^-level concentrations of EDCs in the environment. For example, the concentration of BDE-47 in industrial wastewater is approximately 0.1 μg L^−1^, which is only 1/1630 of its 96 h LC_50_ (0.163 mg L^−1^). Additionally, acute tests focus on lethal effects, which efficiently mask endocrine risks at low concentrations [41,65]. The core hazard of EDCs at environmentally relevant concentrations lies in endocrine disorders and transgenerational transmission caused by long-term exposure. For instance, 0.1 μg L^−1^ of 17β-estradiol increases the proportion of mictic females in *B. plicatilis* by 20–30% and reduces the population growth rate by 10–15%; 250 μg L^−1^ of 4-NP results in a much higher inhibition rate (28%) on the population growth rate of the F_1_ generation of *B. havanaensis* than that of the F0 generation (12%); 80 μg L^−1^ of PFOS decreases the number of eggs per female *B. calyciflorus* from 4.5 to 2.8. Moreover, such effects exhibit a “time-dependent enhancement” characteristic (e.g., exposure to 0.1 μg L^−1^ of BDE-47 for 21 d is required to reduce the population growth rate of *B. plicatilis* by 23%, while no significant effect is observed after 7 d of exposure) [58,65,85]. To integrate these two types of data, a “concentration-effect-time” framework should be established: use LC_50_ to calculate the hazard quotient (HQ) (e.g., Hg^2+^ with an HQ of 0.22 requires priority attention), conduct multi-generational experiments for pollutants with HQ < 1 but chronic effects (e.g., BDE-47 with an HQ of 0.0006), compare the mechanistic differences between high and low concentrations (e.g., BDE-47 induces apoptosis at high concentrations and inhibits reproductive genes at low concentrations), and consider the differences in sensitivity among rotifer species [53,58,101]. In fact, these two types of concentration are complementary. Future studies can simultaneously establish concentration gradients spanning “mg L^−1^ level + ng L^−1^-μg L^−1^ level” and connect molecular and population indicators through genes such as *edh* and *vasa*, thereby providing a complete evidence chain for risk assessment.

## 5. Toxic Mechanisms of Endocrine Disruptors on Rotifers

Distinct EDCs induce diverse toxic effects in rotifers, but oxidative stress is a common underlying mechanism that triggers cascades of damage, including metabolic disruption and reproductive inhibition (Table 2). For example, PBDEs such as BDE-47 and BDE-209 significantly increase ROS levels and suppress SOD and GPx activities [37], impair mitochondrial function and antioxidant capacity, thereby exacerbating ROS accumulation [102]. This leads to lipid peroxidation, membrane damage, and reproductive impairment [101]. BDE-47 also induces lysosomal rupture and cathepsin release, activating caspase-3-dependent oocyte apoptosis (2–3-fold increase) [103], while disrupting energy metabolism by reducing TCA cycle intermediates and inhibiting fatty acid β-oxidation [104]. In addition, PBDEs interfere with hormone-like signaling processes in rotifers—such as pathways involving thyroid hormone-like or estrogen-like molecules—by disrupting TCA cycle intermediates and modulating the expression of β-oxidation enzymes. Functionally, this disruption resembles the interference of PBDEs with vertebrate thyroid/estrogen endocrine pathways [44]; however, rotifers lack specialized endocrine organs (e.g., thyroid glands, ovaries with vertebrate-like hormone synthesis) and rely on localized cellular signaling (e.g., paracrine effects of hormone-like molecules) rather than systemic endocrine regulation. This localized interference ultimately exacerbates metabolic and reproductive dysfunction. PAEs such as DBP and BBP compromise cellular integrity, inducing oxidative and endoplasmic reticulum stress, DNA damage, apoptosis, and neurotoxicity, ultimately inhibiting reproduction [48,49], although the precise molecular network remains unclear. Heavy metals (e.g., Hg^2+^, Cu^2+^) are highly toxic even at low concentrations, promoting ROS overproduction, lipid peroxidation, DNA damage, enzyme inhibition (e.g., Cu^2+^ suppressing antioxidant enzymes), and ionic imbalance (e.g., Ag^+^ disrupting ion homeostasis), leading to feeding inhibition, developmental delays, and transgenerational toxicity [52,53,54]. For phenolic EDCs, 4-NP primarily disrupts energy metabolism and reproductive behaviours. It leads to a reduction in the birth rate and an increase in the mortality rate of *B. havanaensis*, resulting in a significant decline in population growth rate (r). Moreover, long-term exposure impairs the antioxidant enzyme system (e.g., SOD, CAT), triggers oxidative stress, and transmits transgenerational toxicity, ultimately causing reproductive impairment [56]. TCS and TCC both induce ROS production and subsequent DNA double-strand breaks, thereby inhibiting oocyte development in *B. koreanus* [59]. BPA exerts species-specific effects, disrupting neurobehavior in *B. plicatilis* and damaging germ cells, while accumulating transgenerational toxicity in *B. calyciflorus* [61,77]. PFASs impair reproduction via oxidative stress and metabolic interference, potentially altering reproductive strategies [65]. UV-B toxicity is species-specific: oxidative stress and reproductive inhibition dominate in *B. urceolaris* and *B. plicatilis*, whereas feeding inhibition prevails in *B. urceolaris* [79].

The toxicological mechanisms of combined EDC exposure are highly complex, primarily manifesting as three types of mixture effects: concentration addition (CA), independent action (IA), and interaction modification (Table 3). Pollutant types, concentration ratios, and environmental conditions dynamically regulate these effects. Rotifer studies reveal distinct mechanistic patterns. Antagonism in interactions modifies toxicity through competitive target binding, metabolic compensation, or altered bioavailability. For example, in a 1:1 Cu^2+^/Cr^6+^ mixture, Cu^2+^ preferentially binds to membrane transporters, limiting Cr^6+^ uptake, while upregulating metallothionein to chelate Cr^6+^ into non-toxic forms—consistent with “specific antagonism” [54]. Conversely, PSNPs and NaBrO_3_ co-exposure at 1:100 or 100:1 ratios shows “high-concentration adsorption/dispersion antagonism”: excess PSNPs adsorb NaBrO_3_, reducing its bioavailability, while high NaBrO_3_ levels disperse PSNP aggregates, mitigating physical cell damage [89]. Additive effects occur when combined toxicity approximates the sum of the individual toxicities, typically at high concentrations or in non-interacting pathways. In *B. plicatilis* exposed to equitoxic BDE-47 and BDE-209 (1:1) over 72–96 h, rapid metabolism of BDE-209 diminishes interactions, resulting in an IA-like additive effect due to non-overlapping mechanisms [73]. However, high-concentration co-exposure to BDE-47 and DBDPE exceeds metabolic capacity, leading to condition-dependent outcomes: synergism occurs under starvation, whereas toxicity approaches CA model predictions under satiation [40]. Synergism arises when combined toxicity exceeds the sum of the components, driven by cross-activated pathways or enhanced bioaccumulation. For instance, PFOS and PFOA (1:1) co-exposure in *B. calyciflorus* jointly activates oxidative stress and synergistically inhibits TCA cycle enzymes, worsening energy disruption and reproductive inhibition [65]. Similarly, TCS and TCC compete for cytochrome P450 binding sites, increasing mutual bioaccumulation and intensifying DNA damage and apoptosis—aligning with CA’s concentration superposition principle [59]. Notably, mixture effects vary dynamically with exposure duration and environmental factors such as nutritional status [40,73].

In addition, we identified conserved molecular pathways underlying rotifer and mammalian responses to environmental pollutants. In mammals, pathways such as Nrf2-Keap1/ARE and MAPK are integrated with systemic endocrine regulation, including hypothalamic-pituitary-gonadal axis modulation of stress responses [105,106]. The MAPK pathway is well-established in regulating cellular stress, inflammation, and programmed cell death [107]. In contrast, rotifers possess highly homologous antioxidant and stress-response pathways but lack vertebrate-like endocrine axes for reproductive and developmental control. They have no specialized endocrine organs (e.g., hypothalamus, pituitary) and instead rely on localized cellular signals—such as vasa/nanos gene expression and integration of environmental cues—to regulate reproduction. This uncoupling of conserved stress pathways from systemic endocrine regulation highlights rotifers as a unique and complementary model for overcoming limitations of mammalian systems in ecotoxicological studies.

## 6. Integrated Analysis of Transgenerational Toxicity Effects of EDCs on Rotifers

Existing research has clearly demonstrated that the reproductive toxicity of various EDCs on rotifers can be transmitted across generations, showing characteristics of “increased sensitivity in offspring” and “time-dependent accumulation”. The core evidence includes: the inhibition rate of the population growth rate of the F_1_ generation of *B. havanaensis* by 250 μg L^−1^ 4-NP (28%) was significantly higher than that of the F_0_ generation (12%), Ag NPs caused synchronous shortening of the body length of the F_0_ and F_1_ generation larvae of *B. plicatilis*, PSNPs caused developmental delay in the offspring of *B. plicatilis* through maternal egg transfer, and the inhibitory effect of 0.1 μg L^−1^ of BDE-47 on the population growth of *B. plicatilis* became apparent only after 21 d of multi-generation exposure (inhibition rate: 23%), whereas there was no significant effect in the F_0_ generation after 7 d of short-term exposure [41,52,58,90]. The current proposed mechanisms of transgenerational transmission mainly involve three categories: first, direct maternal transfer, such as Ag NPs and PSNPs, which enter the offspring embryos through yolk sac encapsulation or egg membrane permeation and accumulate; second, epigenetic regulation association, as shown by the higher expression inhibition of reproductive-related genes (*vasa*, *nanos*) in the F_1_ generation of *B. plicatilis* exposed to BDE-47 compared to the F0 generation, suggesting intergenerational regulation of gene epigenetic states; third, metabolic programming disorder, such as BDE-47 inhibiting the activity of fatty acid β-oxidase and the levels of key intermediates in the TCA in *B. plicatilis* mothers, leading to energy reserve defects being passed on to the offspring through the maternal metabolic microenvironment [41,52,90,102]. From an ecological risk perspective, such transgenerational toxicity can significantly weaken the natural recovery ability of rotifer populations (e.g., PFOS) reduced the number of eggs produced by a single female of *B. plicatilis* from 4.5 to 2.8 [65]), affect their quality as basic aquatic feed (e.g., shortened larval body length [52]), and may also cause fluctuations in the production of resting eggs by interfering with the reproductive mode transition (e.g., steroid hormones increased the proportion of mixed-sex females of *B. plicatilis* by 20–30% [85]), disrupting the material cycle and food chain structure of aquatic ecosystems. It is urgent to incorporate transgenerational toxicity parameters into ecological risk assessment to improve its accuracy.

## 7. Conclusions and Opinions

This review systematically synthesizes the reproductive toxicity, sensitivity variations, and mechanisms of action associated with PBDEs, PAEs, heavy metals, phenols, PFASs, steroid hormones, and other EDCs (e.g., nanoplastics and disinfection byproducts) in rotifers. It elucidates the potential threats posed by these substances to the fundamental trophic level of aquatic ecosystems (Table 2 and Table 3). All EDCs inhibit rotifer reproduction with distinct toxic effects. Heavy metals (e.g., Hg^2+^, Cu^2+^) are highly toxic at low concentrations, as exemplified by Hg^2+^, which has a 24-h LC_50_ of only 4.51 μg L^−1^ against *B. plicatilis*. PBDEs (e.g., BDE-47) damage ovarian ultrastructure and induce apoptosis. PAEs disrupt lipid metabolism via oxidative stress, and PFASs and steroid hormones alter sexual reproduction ratios. Rotifers exhibit interspecific differences in sensitivity to EDCs, a disparity that is amplified under prolonged exposure conditions. For example, BDE-47 exhibits a 24-h LC_50_ exceeding 22 mg L^−1^ in *B. plicatilis*. In comparison, its 96-h LC_50_ drops to 0.163 mg L^−1^, and *B. havanaensis* is more sensitive to 4-nonylphenol than *P. patulus*. Oxidative stress serves as a universal pathway for toxicity. In contrast, the combined exposure to EDCs results in complex effects. For example, co-exposure to BDE-47 and DBDPE demonstrates synergistic interactions under starvation conditions, whereas it exhibits antagonistic effects when organisms are satiated. Additionally, the interactions between PSNP and bromate depend on the ratio of their concentrations. Significantly, reproductive impairment observed in rotifers facilitates pollutant bioaccumulation and transgenerational transmission, thereby exacerbating ecological risks associated with EDCs.

Despite systematically summarizing advancements in EDC-induced rotifer reproductive toxicity, this review highlights significant limitations that impede practical ecological risk assessment. Current studies lack standardized criteria for selecting rotifer species, test organism life-history stages, exposure durations, and water quality parameters. Notably, there is a marked difference in sensitivity to EDCs between neonates and adults of the test organisms. The short- and long-term LC_50_ values can vary by as much as two orders of magnitude, further exacerbating the variability observed in experimental results. This deficiency results in discrepancies between laboratory-derived toxicity thresholds and the natural responses of rotifers, thereby impeding the accurate extrapolation of laboratory data to risk assessments for aquatic ecosystems. Furthermore, inconsistent experimental protocols complicate comparisons and integration of findings across various research teams. This situation hinders progress in systematic investigations into EDC-induced reproductive toxicity in rotifers. Most studies utilize exposure concentrations (mg L^−1^) that exceed environmentally relevant levels to accelerate the observation of toxic effects. Consequently, there is insufficient data on the long-term sublethal impacts of ng–μg L^−1^ EDC concentrations—consistent with those in actual aquatic environments—on subtle reproductive indicators such as sperm motility and oocyte maturation cycles. This insufficiency hinders accurate low-dose chronic risk assessment. While rotifers serve as optimal models for transgenerational studies, existing research primarily focuses on one to three generations. Investigations into transgenerational effects spanning ten or more generations and their epigenetic regulatory mechanisms remain limited, thereby hindering clarification of cumulative EDC threats to rotifer population survival and patterns of toxicity transmission. The signaling pathways and the invertebrate-specific regulatory mechanisms underlying reproductive inhibition induced by PAE through oxidative stress and endoplasmic reticulum stress remain to be elucidated. PFAS regulation of reproduction-related genes (e.g., *vasa*, *nanos*) and their links to energy metabolism remain unclear, and studies on emerging PFAS alternatives (e.g., GenX, F-53B) are relatively rare. Mammalian toxicity mechanisms (e.g., the Nrf2-Keap1/ARE pathway) are often extrapolated directly to rotifers. This extrapolation fails to account for fundamental differences in reproductive regulation, particularly the alternation between asexual and sexual reproduction, as well as variations in endocrine systems between mammals and rotifers. Furthermore, the gut and surface microbiota of rotifers play a critical role in modulating toxic responses by metabolizing pollutants and regulating host metabolism. However, current research has not addressed the disruption of microbial communities induced by EDCs, nor has it explored the correlations between microbial dysbiosis and reproductive toxicity. This knowledge gap impedes a comprehensive understanding of the underlying toxic mechanisms.

To bridge the existing research gaps, future investigations should integrate advanced technologies to refine and enhance the ecological risk assessment framework. Firstly, it is critical to standardize the selection of model rotifer species, test individual developmental stages (e.g., neonates within 24 h post-hatching), and water quality parameters, and to examine exposure duration gradients. Subsequently, transcriptomics, CRISPR-Cas9 gene editing, and single-cell RNA sequencing should be combined to validate core reproductive pathway functions, thereby reducing data heterogeneity. For emerging PFASs (e.g., GenX) and their degradation products, ultra-performance liquid chromatography-high-resolution mass spectrometry (UPLC-HRMS) can quantify in vivo bioaccumulation levels. Meanwhile, high-throughput assays and proteomics should be integrated to clarify toxicity thresholds. The emphasis should be placed on invertebrate-specific regulatory pathways of PAEs and PFASs to prevent cross-species mechanistic discrepancies. Combined exposure experiments ought to be designed based on the actual ratios of environmental pollutant concentrations. Inductively Coupled Plasma Mass Spectrometry (ICP-MS) and immunofluorescence techniques should be employed to analyze co-accumulation patterns. Furthermore, sublethal endpoints (e.g., resting egg production, population growth rate) should be incorporated, with machine learning applied to develop ecologically relevant risk prediction models. Comparative genomics can investigate conserved stress-response pathways between rotifers and mammals, thereby establishing a validation system involving rotifers, zebrafish, and mammals. Finally, environmental quality criteria for pollutants should be derived from data on rotifer sensitivity and species sensitivity distribution (SSD) models. Ecological thresholds must be formulated by integrating population dynamic models to support revisions of water quality standards and pollution control practices.

## 8. Conclusions

This review follows the PRISMA guidelines and systematically summarizes the research on the reproductive toxicity of endocrine-disrupting chemicals (EDCs) to rotifers. It first clarifies that EDCs, as exogenous substances that interfere with the endocrine systems of organisms, are widely present in aquatic environments. Rotifers, as key live feed in aquaculture and essential species in marine ecosystems, have significant implications for reproductive health. The review covers both traditional EDCs (e.g., polybrominated diphenyl ethers, phthalates, heavy metals, phenols, per- and polyfluoroalkyl substances, and steroid hormones) and emerging EDCs (e.g., microplastics, disinfection by-products, and drug metabolites). It analyzes their specific reproductive hazards to rotifers. Additionally, it compares the sensitivity differences in various rotifer species to EDCs, highlighting oxidative stress as a common toxic mechanism and the context-dependent synergistic or antagonistic effects of combined EDC exposures. Furthermore, it elaborates on how EDCs threaten aquaculture and biodiversity by reducing rotifer population density, altering population structure, and being transferred through the food chain, as well as the impact of transgenerational toxicity on weakening ecosystem resilience. Finally, the review highlights gaps in current research, including data on long-term low-concentration exposure, transgenerational mechanisms, EDC–microbiome interactions, and the toxicity of emerging per- and polyfluoroalkyl substances. It suggests that future research should focus on these areas to provide support for EDC risk assessment and the establishment of water quality standards.

## Figures and Tables

**Table 1:** Summary of the PRISMA Literature Search and Screening Process.

Information Category	Specific Content
Search the database	1. Core databases: CNKI, PubMed; 2. Other sources: Laboratory institutional reports (relevant to 1994).
Exact search terms (including logical combinations)	EDCs, rotifers, reproductive toxicity, toxicity mechanisms.
Publication year	2005–2025 (peer-reviewed literature); 1994 (institutional laboratory reports).
Specific filtering conditions for the database	1. CNKI: Include “Journal Articles/Theses”; exclude “Conference Abstracts/Newspaper Articles”; search terms in “Title/Keywords/Abstract”; language limited to Chinese; 2. PubMed: Filter for “Article/Review”; exclude “Letter/Editorial”; restrict to species “Rotifera”; language limited to English; 3. Laboratory Institution Reports: Include only peer-reviewed formal reports; exclude informal records and unpublished data.
Number of records at each screening stage	1. Initial search results: 689 records from core databases and other sources (CNKI: 215; PubMed: 248; Laboratory institution reports: 3; Other supplementary sources: 223); 2. Number of records after deduplication: 521 (168 duplicates were removed using EndNote X9, including 124 across databases and 44 within the same database but different versions); 3. Number of records retained after initial screening (title/abstract): 172 (349 were excluded, see “Screening Decision Criteria” for details); 4. Number of records retained after full-text screening: 105 (67 were excluded, see “Screening Decision Criteria” for details); 5. Number of records included in the review analysis: 105.
Specific criteria for–screening decisions	I. Inclusion Criteria: 1. Study organisms: Rotifers (Rotifera, e.g., *Brachionus plicatilis*, *Brachionus calyciflorus*); exclude closely related taxa (e.g., cladocerans); 2. Exposure agents: Traditional (PBDEs, PAEs, heavy metals) or emerging EDCs (microplastics, disinfection by-products), with defined exposure conditions (concentration gradients and control groups); 3. Endpoints: Must include reproductive toxicity measures (e.g., egg production, hatching rate, sex ratio, dormant egg output, reproductive gene expression); 4. Data availability: Original data (e.g., LC_50_, mean ± SD) or extractable figures; ≥2 experimental replicates; 5. Publication year: 2005–2025 for peer-reviewed articles; 1994 for laboratory reports. II. Exclusion Criteria: 1. Initial screening (title/abstract): a. Irrelevant topic: No clear link among “rotifers,” “EDCs,” and “reproductive toxicity” (e.g., studies on rotifer growth or EDC effects in fish only); b. Non-research type: Reviews, comments, conference abstracts, patents; non-Chinese/English publications without bilingual abstracts; c. Out-of-range year: Published before 2005 or after 2025 (excluding laboratory reports). 2. Full-text screening: a. Insufficient data: No quantitative reproductive data, missing controls, or <2 replicates; b. Endpoint mismatch: Assesses only acute toxicity (e.g., mortality, immobility) without reproductive metrics; c. Duplicate data: Multiple papers from the same dataset; retain the most recent, comprehensive; d. Poor quality: Flawed design (e.g., non-environmentally relevant concentrations, uncontrolled temperature/salinity); fails CRED minimum standards.
Standards for Research Quality Assessment	Use the combined framework of “ERED guidelines + US EPA toxicity assessment standards”: High quality: Randomized controlled or long-term exposure study, ≥3 replicate groups, complete reproductive toxicity data (e.g., egg production, hatching rate), and statistical analysis; Medium quality: Non-randomized controlled study, 2 replicate groups, complete core reproductive data, no significant data gaps; Low quality: Unclear design, single replicate group, incomplete key data, or no statistical analysis.
Conflict Resolution Process for Reviewers’ Opinions	1. Initial stage: Two independent reviewers conducted literature screening and quality assessment separately, each recording their results; 2. Negotiation stage: Reviewers compared results; any disagreements triggered a cross-check and discussion to identify discrepancies and re-evaluate based on study content; 3. Final decision: If consensus was not reached, a third senior reviewer (with expertise in rotifer endocrine toxicity) was consulted. The final decision followed the majority opinion. A total of 9 conflicts were resolved: 7 through discussion, 2 with third-reviewer input.

**Table 2:** Rotifer sensitivity and toxicity mechanisms to EDCs (Single Exposure).

Category	EDCs	Rotifer	Exposure Duration	Water Quality Parameters	Toxicity Sensitivity (LC_50_)	Action Mechanism	Study Quality Indicators	References
PBDEs	BDE-47	*B. plicatilis*	24 h	Marine, 30 PSU, 20 ± 1 °C, 12L:12D	LC_50_ > 22 mg L^−1^ (24 h) LC_50_ = 2.113 mg L^−1^ (48 h) LC_50_ = 0.376 mg L^−1^ (72 h) LC_50_ = 0.163 mg L^−1^ (96 h)	Oxidative stress, Metabolic disorder, Autophagy, Apoptosis, locomotor inhibition, Feeding inhibition.	Medium	[73,74]
	BDE-47	*B. koreanus*	24 h	Marine, 15 PSU, 25 ± 1 °C, 12L:12D	LC_50_ = 15.7 mg L^−1^ (24 h)	Oxidative stress, Interfere with signaling pathways, Antioxidant.	High	[39]
	BDE-209	*B. plicatilis*	24 h	Marine, 30 PSU, 20 ± 1 °C, 12L:12D	LC_50_ > 120 mg L^−1^ (24 h) LC_50_ = 11.162 mg L^−1^ (48 h) LC_50_ = 1.237 mg L^−1^ (72 h) LC_50_ = 0.295 mg L^−1^ (96 h)	Oxidative stress, locomotor inhibition, Feeding inhibition.	Medium	[73,74]
PAEs	BBP	*B. plicatilis*	24 h	Marine, 30 PSU, 25 ± 1 °C, 12L:12D	LC_50_ = 12 mg L^−1^ (24 h)	Oxidative stress, Endoplasmic Reticulum stress, Reproductive inhibition.	High	[49]
	DBP	*B. calyciflorus*	24 h	Artificial seawater medium according to ISO 6341/OECD 204 [75,76].	LC_50_ = 8.02 mg L^−1^ (24 h)	Oxidative stress, Reproductive inhibition, Developmental retardation.	High	[48]
Heavy Metal	Ag^+^	*B. plicatilis*	24 h	Artificial seawater medium according to ISO 6341/OECD 204.	LC_50_ = 18.7 mg L^−1^ (24 h) LC_50_ = 3.4 mg L^−1^ (48 h)	Feeding inhibition, Developmental retardation, Transgenerational toxicant accumulation.	High	[52]
	Hg^2+^	*B. plicatilis*	24 h	Marine, 30 PSU, 25 ± 1 °C, 12L:12D	LC_50_ = 4.51 µg L^−1^ (24 h)	Oxidative stress, Reproductive and developmental inhibition.	Medium	[53]
	Cd^2+^	*B. plicatilis*	24 h	Marine, 30 PSU, 25 ± 1 °C, 12L:12D	LC_50_ = 2.07 mg L^−1^ (24 h) LC_50_ = 2.89 mg L^−1^ (24 h)			
	Pb^2+^	*B. plicatilis*	24 h	Marine, 30 PSU, 25 ± 1 °C, 12L:12D	LC_50_ = 2.83 mg L^−1^ (24 h)			
	Cu^2+^	*B. calyciflorus*	24 h	Freshwater, EPA medium, 25 ± 1 °C, 12L:12D	LC_50_ = 6.2 µg L^−1^ (24 h)	Oxidative stress, Enzyme inhibition, Reproductive inhibition.	High	[54]
	Zn^2+^	*B. calyciflorus*	24 h	Freshwater, EPA medium, 25 ± 1 °C, 12L:12D	LC_50_ = 12.62 mg L^−1^ (24 h)			
	Cr^6+^	*B. calyciflorus*	24 h	Freshwater, EPA medium, 25 ± 1 °C, 12L:12D	LC_50_ = 17.29 mg L^−1^ (24 h)			
	Mn^2+^	*B. calyciflorus*	24 h	Freshwater, EPA medium, 25 ± 1 °C, 12L:12D	LC_50_ = 67.32 mg L^−1^ (24 h)			
Phenols	4-NP	*B. havanaensis*	24 h	Freshwater, EPA medium, 25 ± 1 °C, 16L:8D	LC_50_ = 250 µg L^−1^ (24 h)	Oxidative stress, Metabolic obstruction, Enzyme inhibition, Reproductive inhibition, Transgenerational toxicant accumulation.	High	[58]
		*P. patulus*	24 h	Freshwater, EPA medium, 25 ± 1 °C, 16L:8D	LC_50_ = 500 µg L^−1^ (24 h)			
	TCS	*B. koreanus*	24 h	Marine, 15 PSU, 25 ± 1 °C, 12L:12D	LC_50_ = 393.1 µg L^−1^ (24 h)	Oxidative stress, DNA damage, Reproductive inhibition.	High	[59]
	TCC	*B. koreanus*	24 h	Marine, 15 PSU, 25 ± 1 °C, 12L:12D	LC_50_ = 338.1 µg L^−1^ (24 h)	Oxidative stress, DNA damage, Reproductive inhibition.		
	BPA	*B. plicatilis*	24 h	Marine, 15 PSU, 25 ± 1 °C, 12L:12D	LC_50_ > 22 mg L^−1^ (24 h)	Oxidative stress, Neurobehavioral interference.	High	[77]
		*B. calyciflorus*	24 h	Freshwater, EPA medium, 25 ± 1 °C, 12L:12D	LC_50_ = 13.76 mg L^−1^ (24 h)	Reproductive inhibition, Transgenerational toxicant accumulation.	High	[61]
PFAS	PFOS	*B. calyciflorus*	24 h	Artificial seawater medium according to ISO 6341/OECD 204.	LC_50_ = 61.8 mg L^−1^ (24 h)	Oxidative stress, Reproductive inhibition.	High	[65]
	PFOA	*B. calyciflorus*	24 h	Artificial seawater medium according to ISO 6341/OECD 204.	LC_50_ = 150 mg L^−1^ (24 h)	Increased microcapsule rate (resting egg), Metabolic interference.		
Others	UV-B	*B. asplanchnoidis*	24 h	Brackish, 10 PSU, 20 ± 0.5 °C, 16L:8D	LD_50_ = 28.53 kJ/m^2^ (24 h)	Oxidative stress, DNA damage.	High	[78]
		*B. urceolaris*	24 h	Marine, 30 PSU, 280–320 nm, 12L:12D	LD_50_ = 5.856 kJ/m^2^ (24 h) LD_50_ = 4.516 kJ/m^2^ (48 h) LD_50_ = 1.730 kJ/m^2^ (96 h)	Feeding inhibition.	Medium	[79]
		*B. plicatilis*	24 h	Marine, 30 PSU, 280–320 nm, 12L:12D	LD_50_ = 4.393 kJ/m^2^ (24 h) LD_50_ = 2.694 kJ/m^2^ (48 h) LD_50_ = 1.720 kJ/m^2^ (96 h)	Oxidative stress, Reproductive inhibition.		
	C_2_H_3_BrNO	*B. calyciflorus*	24 h	Freshwater, EPA medium, 25 ± 1 °C, 16L:8D	LC_50_ = 2.46 mg L^−1^ (24 h)	Oxidative stress, Transition from parthenogenesis to sexual reproduction.	High	[80]
	C_2_H_4_ClNO	*B. calyciflorus*	24 h	Freshwater, EPA medium, 25 ± 1 °C, 16L:8D	LC_50_ = 12.49 mg L^−1^ (24 h)			
	C_2_H_3_Cl_2_NO	*B. calyciflorus*	24 h	Freshwater, EPA medium, 25 ± 1 °C, 16L:8D	LC_50_ = 223.12 mg L^−1^ (24 h)			
	APAP	*B. rotundiformis*	96 h	Marine, 15 PSU, 25 ± 1 °C, 12L:12D	LC_50_ > 5000 mg L^−1^ (96 h)	Oxidative stress, Reproductive inhibition.	High	[81]
	OTC	*B. rotundiformis*	96 h	Marine, 15 PSU, 25 ± 1 °C, 12L:12D	LC_50_ > 200 mg L^−1^ (96 h)			
	TPTCl	*B. koreanus*	24 h	Marine, 15 PSU, 25 ± 1 °C, 12L:12D	LC_50_ = 29.6 µg L^−1^ (24 h)	Oxidative stress, Reproductive and developmental inhibition.	High	[82]

Note: Among them, BDE-47 is 2,2′,4,4′-tetrabromodiphenyl ether; BDE-209 is 2,2′,3,4,4′,5′,6-heptabromodiphenyl ether; BBP is butyl benzyl phthalate; DBP is dibutyl phthalate; 4-NP is 4-nonylphenol; TCS is triclosan; TCC is triclocarban; BPA is bisphenol A; PFOS is perfluorooctanesulfonic acid; PFOA is perfluorooctanoic acid; UV-B is Ultraviolet B; C_2_H_3_BrNO is 2-bromoacetamide; C_2_H_4_ClNO is 2-chloroacetamide; C_2_H_3_Cl_2_NO is 2,2-dichloroacetamide; APAP is paracetamol; OTC is oxytetracycline; TPTCl is triphenyltin chloride. The different studies cited for the action mechanism in each column are all derived from studies on the same rotifer species. Study quality was graded as High/Medium/Low using a combined framework of the CRED guidelines and EPA toxicity study assessment criteria.

**Table 3:** Rotifer sensitivity and toxicity mechanisms to EDCs (Combined exposure).

Syndication Kinds	Rotifer	Mixing Ratio Method	Toxic Action	Study Quality Indicators	Reference
BDE-47 + BDE-209	*B. plicatilis*	Concentration (or toxicity) ratio 1:1	Interaction modification (early synergy/late weak antagonism); late near independent action (IA).	High	[73]
BDE-47 + DBDPE	*B. plicatilis*	Concentration ratio 1:1	Interaction modification (nutrition state-dependent synergy/antagonism); high concentration close to concentration addition (CA).	High	[40]
Cu^2+^ + Cr^6+^	*B. calyciflorus*	Toxicity ratio 1:1	Interaction correction (specific antagonism).	High	[54]
Cu^2+^ + Zn^2+^	*B. calyciflorus*	Concentration ratio 1:1	Interaction correction (competitive antagonism).		
Cu^2+^ + Cd^2+^	*B. calyciflorus*	Concentration ratio 1:1	Interaction correction (metal binding site competition antagonism).		
Zn^2+^ + Cd^2+^	*B. calyciflorus*	Concentration ratio 1:1	Interaction correction (synergistic superposition of toxic pathways).		
Cu^2+^ + Zn^2+^ + Cd^2+^	*B. calyciflorus*	Toxicity ratio 1:1:1	Interaction correction (synergistic effect of multi-metal oxide stress).		
Cu^2+^ + Zn^2+^ + Cr^6+^	*B. calyciflorus*	Toxicity ratio 1:1:1	Interaction correction (oxidative damage + enzyme inhibition synergy).		
Cu^2+^ + Cd^2+^ + Mn^2+^	*B. calyciflorus*	Toxicity ratio 1:1:1	Interaction correction (antioxidant antagonism mediated by Mn^2+^).		
Cu^2+^ + Zn^2+^ + Cd^2+^ + Cr^6+^	*B. calyciflorus*	Toxicity ratio 1:1:1:1	Interaction correction (antagonism of multi-metal competitive binding).		
Cu^2+^ + Zn^2+^ + Cd^2+^ + Cr^6+^ + Mn^2+^	*B. calyciflorus*	Toxicity ratio 1:1:1:1:1	The interaction correction (synergistic burst of oxidative stress after depletion of Mn^2+^).		
Cu + Hg, As + Pb, Cd + Pb	*P. similis*	Toxicity ratio 1:1	Interaction correction (cooperative entry of heavy metal ion channels).	High	[91]
As + Hg, Zn + Pb, Cu + Cd	*P. similis*	Toxicity ratio 1:1	Interaction correction (competitive antagonism of metal transporters).		
PFOS + PFOA	*B. calyciflorus*	Concentration ratio 1:1	Interaction modification (peroxisome activation pathway synergy); close to concentration addition (CA).	High	[65]
TCS + TCC	*B. koreanus*	Concentration ratio 1:1	Interaction modification (CYP450 enzyme competitive inhibition synergy); close to concentration addition (CA).	High	[59]
ETE + LEV	*A. fissa*	Mixed concentrations of 31.25/6.25 µg L^−1^	No comparison	High	[86]
	*B. calyciflorus*	Mixed concentrations of 250/50 µg L^−1^	No comparison		
PSNPs + NaBrO_3_	*B. calyciflorus*	Concentration ratio 1:1	Interaction correction (carrier-enhanced bioaccumulation synergy).	High	[89]
	*B. calyciflorus*	concentration ratio of 1:100 or 100:1	Interaction correction (antagonism of high-concentration adsorption/dispersion effect).		

Note: Among them, BDE-47 is 2,2′,4,4′-tetrabromodiphenyl ether; BDE-209 is 2,2′,3,4,4′,5′,6-heptabromodiphenyl ether; DBDPE is decabromodiphenyl ethane; PFOS is perfluorooctanesulfonic acid; PFOA is perfluorooctanoic acid; TCS is triclosan; TCC is triclocarban; ETE is ethinylestradiol; LEV is levonorgestrel; PSNPs is polystyrene nanoplastics; NaBrO_3_ is Sodium bromate. Study quality was graded as High/Medium/Low using a combined framework of the CRED guidelines and EPA toxicity study assessment criteria.

## Data Availability

No data was used for the research described in the article.
